# Tetracycline‐induced black hairy tongue

**DOI:** 10.1002/jgf2.300

**Published:** 2020-01-30

**Authors:** Kota Sakaguchi, Takashi Watari

**Affiliations:** ^1^ Community Care Unnan City Hospital Shimane Japan; ^2^ Postgraduate Clinical Training Center Shimane University Hospital Shimane Japan

**Keywords:** antibiotics, black hairy tongue, drug‐induced, oral hygiene, tetracycline

## Abstract

A blackish discolouration on the central part of the dorsal tongue in the front of the circumvallate papillae.

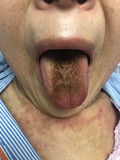

A 73‐year‐old woman with a history of hypertension presented to our department with persistent high fever of 38°C and general fatigue for about 2 weeks before her visit. Except her fever, all other vital signs were normal. Physical examination revealed lymphadenopathy of the postcervical lymph node and a rash on the anterior chest. Blood tests showed elevated liver enzymes and elevated inflammatory markers (C‐reactive protein, 30 mg/dL). In addition, we found an eschar on the posterior neck and suspected either Japanese spotted fever or tsutsugamushi disease; hence, we administered minocycline (200 mg/day). In 2 days, the rash reduced and the fever and general fatigue disappeared. The tetracycline (minocycline) course was continued for 7 days, and she recovered completely before discharge.

However, on follow‐up examinations 5 days after discharge, she reported discoloration on her tongue (Figure 1). Because there were no other causes other than those described below, the blackish finding was diagnosed as black hairy tongue. In this condition, the filiform papillae on the dorsum of the tongue become hypertrophic, elongated, and show horny projections, appearing hair‐like, with black to yellow discoloration.^1^ Black hairy tongue has a prevalence of about 0.5% among adults, although the prevalence varies depending on the population studied. The disease affects men (18%) nearly three times more than women (6%).^2^ Although the exact etiology of hairy tongue is unknown, few possible contributory factors include smoking, poor oral hygiene, and administration of antibiotics (penicillin and tetracyclines).^3^ Systemic diseases, such as HIV and malignancy, have also been linked to hairy tongue.^4^ Symptoms include dysgeusia and nausea, though they are very rare. Natural remission of this condition in a hygienic oral environment is common and aided by the therapeutic efficacy of treating the associated systemic diseases.

**Figure 1:**
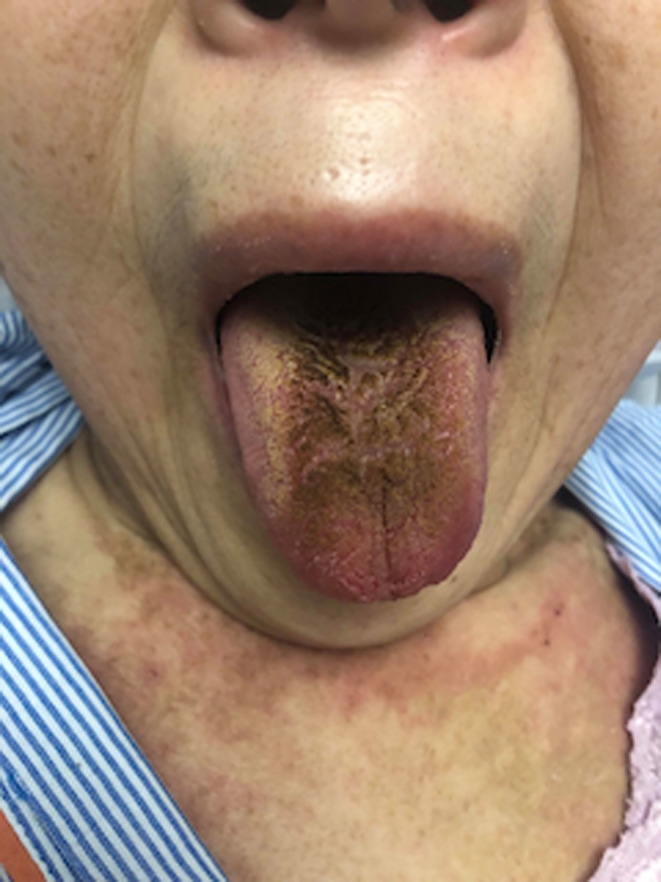
A blackish discoloration on the central part of the dorsal tongue in the front of the circumvallate papillae

In the present case, the onset of hairy tongue occurred 5 days after the discontinuation of antibiotic treatment, suggesting that the etiologic factor could be tetracycline. We advised the patient to maintain oral hygiene and subsequently observed improvement of the condition of her tongue. Although black hairy tongue is rare and self‐limiting, it is important to consider this drug‐induced cause when diagnosing and treating similar conditions, along with a thorough medical history and physical examination.

## CONFLICT OF INTEREST

The authors have stated explicitly that there are no conflicts of interest in connection with this article.

## AUTHOR CONTRIBUTION

All authors had access to the information used; all authors participated in the preparation of this manuscript; and all authors read and approved the final version of the manuscript.
