# Supplementary material for: Tetracycline‐induced black hairy tongue
Source: J Gen Fam Med. 2020 Jan 30;21(3):80–1. doi: 10.1002/jgf2.300 (PMC7260164; doi:10.1002/jgf2.300)
Supplement: Supplementary file 1 [file JGF2-21-80-s001.pdf]

COI Self-Report Form: Journal of General and Family Medicine, an official Journal of Japan Primary Care Association

All Authors' Name: Kota Sakaguchi / Takashi Watari

Title of the paper: Tetracycline-induced black hairy tongue

All authors must describe the COI status, within one year before the paper submission, with any companies, organizations, and/or groups related to the content of the publication. The Corresponding Author should guarantee that he/ she has obtained all potential Conflict of Interest from all authors and must disclose them in the table below.

Item	Applicability	If "Yes," please list the name of the author and the name of all related entities.
(1) Assuming a position of a board member or advisor in a profit-making business, legal entity, or organization run by you, your spouse, or your first-degree relative<br>1 million yen or more annual compensation from a single business, entity, or group	No	
(2) Stock holdings<br>1 million yen or more annual dividends or 5% or more interests in the applicable business	No	
(3) Patent royalties<br>1 million yen or more annual income per patent	No	
(4) Honoraria for lectures<br>1 million yen or more total annual income from a single company or organization	No	
(5) Honoraria for manuscripts<br>500 thousand yen or more total annual income from a single company or organization<br>annual income from a single business or group	No	
(6) Total clinical research grants (e.g. commissioned research, joint research)<br>2 million yen or more total annual research grants paid from a single company or organization to you or your department	No	
(7) Total scholarship grants (incentives), etc.<br>2 million yen or more total annual scholarship contributed by a single company or organization to you or your department	No	
(8) Courses endowed by companies, etc.<br>(Fill in if you belong to any course endowed by a company, etc.)	No	
(9) Receiving travel expenses or gifts<br>50 thousand yen or more annually from a single company or organization	No	

(This COI report will be kept for two years after the publication of the paper. The content of your report will not be disclosed to a third party.)

Corresponding author's name Kota Sakaguchi

Signature Kota Sakaguchi Date 2019/6/18

CONSENT FORM FOR CASE REPORTS

For a patient's consent to publication of information about them in a journal or thesis

Name of person described in article or shown in photograph: Kimie Okada

Subject matter of photograph or article: Black hairy tongue

Title of article: Tetracycline-induced black hairy tongue

Medical practitioner or corresponding author: Takashi Watari

I Kimie Okada [insert full name] give my consent for this information about MYSELF OR MY CHILD OR WARD/MY RELATIVE [insert full name]:____________________, relating to the subject matter above ("the Information") to appear in a journal article, or to be used for the purpose of a thesis or presentation.

I understand the following:

1. The Information will be published without my name/child's name/relatives name attached and every attempt will be made to ensure anonymity. I understand, however, that complete anonymity cannot be guaranteed. It is possible that somebody somewhere - perhaps, for example, somebody who looked after me/my child/relative, if I was in hospital, or a relative - may identify me.
2. The Information may be published in a journal which is read worldwide or an online journal. Journals are aimed mainly at health care professionals but may be seen by many non-doctors, including journalists.
3. The Information may be placed on a website.
4. I can withdraw my consent at any time before online publication, but once the Information has been committed to publication it will not be possible to withdraw the consent.

Signed: 岡田キミエ Date: 2019.6.18

Signature of requesting medical practitioner/health care worker:

Kota Sakaguchi Date: 2019/6/18
